# Drug-Resistant Tuberculosis Treatment Outcomes among Children and Adolescents in Karachi, Pakistan

**DOI:** 10.3390/tropicalmed7120418

**Published:** 2022-12-06

**Authors:** Amyn A. Malik, Uzma Khan, Palwasha Khan, Aliya Anwar, Naseem Salahuddin, Saira Khowaja, Aamir J. Khan, Salman Khan, Hamidah Hussain, Farhana Amanullah

**Affiliations:** 1 Interactive Research and Development (IRD) Global, Singapore 049145, Singapore; 2 London School of Hygiene & Tropical Medicine, London WC1E 7HT, UK; 3 The Indus Hospital and Health Network, Karachi 75190, Pakistan; 4 Department of Global Health and Social Medicine, Harvard Medical School, Boston, MA 02115, USA; 5 Center for Global Health Delivery, Harvard Medical School, Boston, MA 02115, USA; 6 Communicable Diseases Control, Department of Health, Government of Sindh, Hyderabad 65320, Pakistan

**Keywords:** drug-resistant TB, children, adolescents, treatment outcomes, Pakistan

## Abstract

Background: Significant data gaps exist for children and adolescents with drug-resistant (DR) TB, particularly from high TB incidence settings. This report provides a descriptive analysis of programmatic outcomes among children and adolescents treated for DR-TB in Pakistan. Methods: We extracted programmatic data from January 2014 to December 2019 from a tertiary care hospital with specialised child and adolescent DR-TB services. A physician assessed all children and adolescents (0–19 years) with presumptive DR-TB, including details of exposure to DR-TB, medical history, radiology, and laboratory results. All patients received treatment as per national DR-TB management guidelines based on WHO recommendations. Results: There were 262 treatment episodes for 247 patients enrolled during the study period. The median age of the cohort was 16 years (IQR: 13–18 years) with 16 (6.1%) children being under 5 years; 237 (90.5%) patients had pulmonary TB. The majority of the patients (194 or 74.1%) experienced a favourable treatment outcome and 26 (9.9%) died while on treatment. Female patients (78.5%) were more likely to experience favourable outcomes compared to males (64.7%; chi-sqr *p*-value = 0.02). Conclusions: We found high rates of favourable outcomes in children and adolescents treated for DR-TB. However, there were few young children in our cohort and there was a considerable gender gap that enhanced efforts to diagnose DR-TB in young children and to elucidate and mitigate the reasons for poor outcomes amongst males.

## 1. Background

TB is the second leading infectious cause of death globally after COVID-19. It led to approximately 10.6 million new patients and 1.6 million deaths in 2021 [1]. Approximately, 450,000 TB patients annually have rifampicin-resistant (RR)/multi-drug resistant (MDR) TB [1]. Treatment using second-line drugs is long, complex, and toxic, with an average treatment success of 60%.

In 2021, children accounted for approximately 11% of all patients with TB but accounted for 14% of mortality due to TB [1]. Drug-resistant tuberculosis (DR-TB) is a major public health problem affecting children and adolescents, with the highest risk in household contacts of adults diagnosed with DR-TB [2,3]. Approximately 30,000 children develop MDR-TB annually. A recent systematic review noted that 13.6% of all children with TB may have some form of DR-TB with the pooled proportion of MDR-TB being 3.7% (95% CI, 3.5–4.0%). There was a lot of regional heterogeneity with the proportion of MDR-TB in high-income countries being 1.8% [4]. Most children have primary DR-TB rather than acquired resistance. Studies have shown that proportion of children with MDR-TB amongst children with TB is similar to adults [3]. 

Paucibacillary disease in younger children combined with a likelihood to develop severe forms of the disease, such as miliary TB and TB meningitis, complicates diagnosis, and management. Drug susceptibility testing results are often lacking for children as a diagnosis is made on clinical grounds as young children often cannot expectorate sputum for bacteriological diagnosis and testing [5] This can result in treatment being started empirically, often based on the drug susceptibility profile of the household contact presumed to be the index patient. 

The cumulative risk of TB increases with age as exposure accumulates [6], and the clinical presentation also changes. Presentation of DR-TB in adolescence varies from that in early childhood with older children having parenchymal infiltrates and more frequent cavitation [7]. This requires differentiated care, yet adolescents, generally, have not been recognised as a distinct population by guidelines and national TB programmes [8]. Although the principles of treatment for DR-TB in children are similar to adults, with the use of four to five effective drugs [9], more evidence needs to be produced to understand the demographics, clinical characteristics, and treatment outcomes among children and adolescents diagnosed and treated for DR-TB to further inform policy and practice.

Pakistan is one of the eight countries that contribute approximately 68% of the global TB burden with an estimated 611,000 TB patients, including 36,000 MDR/RR-TB patients annually [1]. Pakistan is a high DR-TB burden country ranking fourth globally. The highest numbers of RR/MDR-TB patients are reported in the province of Punjab (46%), followed by Sindh (39%), Khyber Pakhtunkhwa (10%) and Baluchistan (2.7%) [10]. Treatment success for RR/MDR/-TB patients who started treatment in 2019 was 73% and for pre-XDR/XDR-TB patients was 67% [1].

Significant data gaps exist for children and adolescents affected with DR-TB, particularly from high TB incidence settings, where they may comprise 1/3 to half of the population. In this report, we provide a descriptive analysis of programmatic outcomes among children and adolescents treated for DR-TB at a tertiary care hospital in Karachi, Pakistan between 2014 and 2019. 

## 2. Methods

### 2.1. Study Setting

This study was carried out at The Indus Hospital, Karachi, Pakistan. Karachi is the largest city in Pakistan located in the province of Sindh, with a population of approximately 20 million. The Indus Hospital is one of four public sector clinical sites for DR-TB treatment in the city and offers a community-based DR-TB treatment program. The annual number of DR-TB patients reported from Sindh was 1184 in 2019 and the treatment success rate for the 2017 cohort was 58%. The annual number of DR-TB patients reported from Karachi is approximately 400.

We used de-identified retrospective surveillance data collected at a tertiary referral DR-TB treatment site in Karachi, Pakistan, from January 2014 to December 2019. All testing and treatment services including social and nutrition support were offered free of cost at the study site. This study site reported a third of all children diagnosed with DR-TB from Pakistan to the WHO in 2019.

### 2.2. Patient Recruitment and Enrollment at Site

Confirmation of DR-TB diagnosis was based on a combination of Xpert MTB/RIF assay, Mycobacterium TB (M.TB) Mycobacteria Growth Indicator TB (MGIT) culture and Drug sensitivity testing (DST), line probe assay (LPA), and/or clinical diagnosis. Physicians trained to manage DR-TB in children and adolescents carried out an assessment of all children and adolescents (0–19 years) referred with presumptive DR-TB. This typically included a detailed exposure history, medical history, a clinical examination, and conducting routine radiology and laboratory investigations at the start of DR-TB treatment. The site transitioned to an all-oral regimen (including for children and adolescents) in early 2017 for the treatment of DR-TB.

Prior to 2017, each DR-TB patient was started on an conceptualized longer treatment regimen (LTR) based on their DST results. The LTR for a patient with no resistance to any second-line drug (SLD) was comprised of at least 8 months of treatment with amikacin (Am)/kanamycin (Km)/capreomycin (Cm)  +  levofloxacin (Lfx)  +  ethionamide (Eto)  +  cycloserine (Cs)  +  pyrazinamide (Z) and 12 months treatment with Lfx + Eto + Cs + Z. For patients with resistance to any SLD, para-aminosalicylic acid (PAS) was added to the regimen. Patients on LTR were treated for at least 20 months. Once bedaquiline and delamanid became available in 2017, eligible children were treated with an all-oral LTR regimen where bedaquiline or delamanid replaced the injectable agent in the regimen (Am/Km/Cm).

### 2.3. Data Extraction and Analysis

All patients aged 0–19 years at the site were registered on the Electronic Nominal Registration System (ENRS) database, which is a Microsoft Excel-based recording and reporting system maintained at all DR-TB treatment sites in the country. De-identified data were extracted for analysis for this study with DR-TB outcomes declared according to the National TB Program (NTP) definitions. Data access was restricted to the study investigators. Outcomes were grouped into favourable (cured/treatment completed) or unfavourable (died/failed treatment/lost to follow-up/not evaluated). Drug resistance type was defined as follows [11]:

(1) RR/MDR-TB: resistance to Rifampicin or both Rifampicin and Isoniazid
(2) Pre-XDR-TB: RR/MDR-TB plus resistance to a fluoroquinolone
(3) XDR-TB: Pre-XDR-TB plus resistance to any injectable and a fluoroquinolone
(4) Other: Not meeting the definition of RR/MDR-TB, Pre-XDR-TB, or XDR-TB
(5) Suspected DR-TB: not having a microbiological confirmation but resistance is suspected based on clinical symptoms, and positive exposure or contact history.

Descriptive analysis was carried out and frequency tables were created to determine clinical and demographic distributions of children and adolescents treated for DR-TB. Patients were stratified into four age groups as follows: 0–4 years, 5–9 years, 10–14 years, and 15–19 years. The Chi-squared test was used to assess the bivariate association between categorical demographic characteristics and DR-TB outcomes. The Wilcoxon rank-sum test was used to assess the bivariate association between continuous variables and DR-TB treatment outcomes. All analysis was carried out on Stata (Version 16, StataCorp LP, College Station, TX, USA).

### 2.4. Ethics Statement

The Institutional Review Board (IRB) at Interactive Research and Development (IRD) approved this study (IRD_IRB_2021_02_007). 

## 3. Results

**Figure 1:**
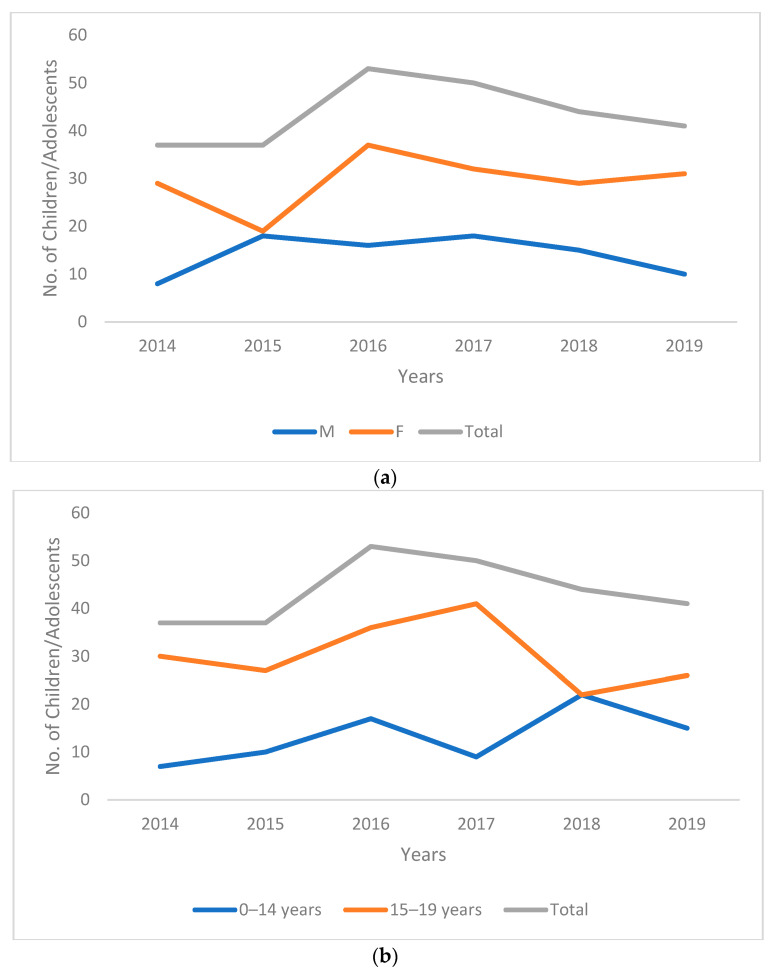
(**a**) Annual enrollment by gender among child and adolescent DR-TB patients at a tertiary care hospital in Karachi, Pakistan between January 2014 and December 2019. (**b**) Annual enrollment by age groups amongst child and adolescent DR-TB patients at a tertiary care hospital in Karachi, Pakistan between January 2014 and December 2019.

Between 2014 and 2019, the site treated 247 children and adolescents, of which 15 patients were treated twice as they had either failed treatment or were lost to follow-up, giving a total of 262 treatment episodes. Of these 247 children and adolescents, 116 (47%) had a history of previous TB treatment. One child tested positive for HIV. Figure 1a shows the yearly treatment enrollment for the site by gender. There were more females (*n* = 177, 67.6%) as compared to males (*n* = 85, 32.4%) overall with a male-to-female ratio of 0.48. The annual enrollment ranged from 37 to 53 with the highest number of patients enrolled in 2016. More female patients were enrolled each year as compared to males with the male-to-female ratio ranging from 0.28 to 0.95 (highest in 2015). 

The median age of the cohort was 16 years (IQR:13–18 years) with 16 (6.1%) children being under 5 years, 10 (3.8%) children between 5 and 9 years of age, and 54 (20.6%) children between 10 and 14 years of age. Figure 1b shows the yearly enrollment by age groups with more patients 15–19 years of age being treated each year. In 2018, the number of patients 0–14 years and 15–19 years was equal. Of the 262 patients, 237 (90.5%) had pulmonary TB while three (1.1%) had both pulmonary and extra-pulmonary TB. All patients treated twice had pulmonary TB. The resistance pattern observed in this cohort is detailed in Table 1. Most patients had RR/MDR-TB (*n* = 198; 75.6%). 

**Figure 2:**
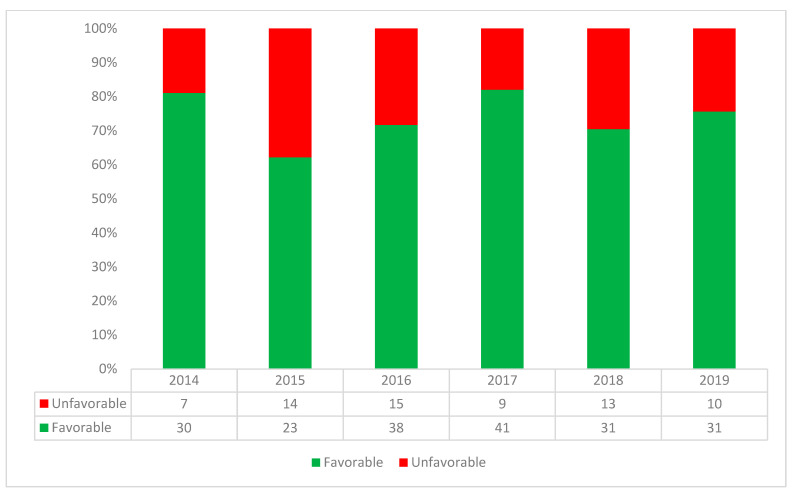
Annual treatment outcomes amongst children and adolescents receiving DR-TB treatment at a tertiary care hospital in Karachi, Pakistan between 2014 and 2019.

Of the 262 individuals, 194 (74.1%) experienced a favourable treatment outcome, 26 (9.9%) died while on treatment, 20 (7.6%) failed treatment, 19 (7.3%) were lost to follow-up, and three (1.2%) were not evaluated. All not evaluated children were 15–19 years of age. The child positive for HIV died while on TB treatment. Favourable outcomes ranged between 68.7% and 80.0% across the four age groups. Children under 5 years were less likely to have favourable outcomes (68.7%) but the difference was not statistically significant (Table 2). Children 5–9 years of age had the most favourable outcomes. There was statistically no difference in treatment outcomes by type of resistance ranging between 64.3% and 75.8% (Table 1). Of the 198 children and adolescents with bacteriologically confirmed RR/MDR-TB, 75.8% (150) experienced favourable outcomes. However, there was a difference in the outcomes by gender with female patients (139/177, 78.5%) experiencing more favourable outcomes compared to males (55/85, 64.7%; chi-sqr *p*-value = 0.02). Males experienced more unfavourable outcomes amongst all age groups including among 10–19 year-olds. Favourable outcomes across the 5-year period ranged from 62% to 82% as shown in Figure 2 with favourable outcomes being highest in 2017.

## 4. Discussion

We report findings on 262 children and adolescents treated over a 5-year study period at a single site in Karachi. Favourable treatment outcomes were observed among 74% of all children and adolescents treated which compares well to the outcomes reported by Harausz et al. [12] and Tola et al. [13] in two pediatric DR-TB meta-analyses and are better than those recently reported in the region [14] However, favourable outcomes are lower than those reported from a multi-center study from Pakistan which reported a success rate of approximately 82% in a younger cohort of children less than 15 years old [15]. Abubakar et al. recently reported a treatment success rate of 50% among children with pulmonary XDR-TB [16]. 

Drug-resistant TB in children is underdiagnosed and undertreated globally with a huge detection gap of 82% [1]. The adolescent subgroup of 10–19 years is not described separately as data reported to WHO is disaggregated as 0–4 years, 5–14 years, and 15–24 years thus masking the specific epidemiology and outcomes of children and adolescents (esp. 15–19 years). Our study provides important insight into drug-resistant TB outcomes in children as well as adolescents 10–19 years, a neglected yet vulnerable subgroup [8,17,18]. 

The overall outcomes indicate favourable outcomes among 74% of all children and adolescents treated over 5 years, with a drop in treatment success rates among individuals with confirmed pre-XDR (70%) and XDR TB (67%). Our cohort had more females (68%) overall and consistently for all age categories. The female preponderance among young TB patients is also seen in our drug-sensitive TB patient cohort reported earlier by Hamid et al. [19] and in the multicenter study on DR-TB in children from Pakistan [15]. Females were more likely to experience better outcomes compared to males (79% vs. 65%) overall and across all age groups. These results are in contrast to those from the earlier study in Pakistan that did not show any difference in outcome by gender [15]. Our findings continue to raise questions that require urgent study, regarding under-diagnosis and poor access to care among boys and young men in our setting and the causes of poorer treatment outcomes in this group. A larger proportion of girls and young women have been detected with TB in our program as reviewed by Codlin et.al. who reported a male-to-female ratio of 0.45 in the <15 years age group and 0.82 among the 15–24 year age group and also reported a similar trend in the overall TB data from Pakistan [20]. A similar gender proportion was reported in India recently for children and adolescents with DR-TB [14]. The poorer outcomes among very young children (especially boys) found in our cohort are consistent with findings among younger children affected with DS-TB in this population [19] and may be associated with delayed health seeking for male children and underlying malnutrition resulting in higher mortality rates. Adolescent boys in our program also experienced more unfavourable outcomes (higher mortality and treatment failure) compared to girls. This higher risk among males is seen in young adults in other settings; however, the underlying reasons remain unclear and may include factors resulting in undiagnosed TB in male adolescents who may be less likely to access healthcare and adhere to treatment and thus more likely to fail treatment [21,22,23]. There is an urgent need to evaluate gender-based and age-disaggregated differences in care-seeking behavior, patient pathway analyses and barriers to care especially among young and adolescent boys and develop and implement programmatic interventions that address these barriers.

Although adolescents (10–19 years) are at a high risk for TB disease, research related to this age group is limited. Most studies divide patients as children (<15 years) or adults, and hence there are limited data to describe the characteristics of adolescent DR-TB. Adolescents develop more transmissible, adult forms of disease and have an increased potential for transmission given higher social mixing. They comprise 84% of our cohort. This age group experienced mostly favourable outcomes (74%) similar to the findings by Tierney et al. (76%) and in contrast to Moyo et al. who reported high rates of loss to follow-up (43%) contributing to poorer outcomes [18,24] Of note, our study had many more adolescent females compared to the Peru cohort (68% vs. 42%) [24]. Adolescents with TB generally have unique challenges including late presentation, high rates of loss to follow-up, and potential for underdosing during treatment especially during DR-TB treatment [25,26,27]. They can often present with severe disease in terms of cavitation on chest X-ray and sputum smear positivity [28]. A recent qualitative study from Mumbai, India documented that adolescents experience a disruption of social life due to the disease and treatment. They experience emotional trauma and treatment fatigue/burnout during the treatment and some of this can be associated with non-adapted healthcare service provision. The study noted differences in the experiences of adolescents and children during DR-TB treatment including around treatment adherence and interruption, which was reported more commonly by adolescents [29]. Hence, they need to be conceptual as a separate population and their characteristics are described as such. The provision of adolescent-friendly services and age-specific counselling can also help improve outcomes for this age segment. 

Although we did not find any statistically significant difference in terms of outcomes between the age categories, the youngest group (0–4 years, 7% of our cohort) experienced slightly worse outcomes, likely related to more severe forms of TB and lower immunity in the 0–4 age group, whereas the 5–9 year age group had the best outcomes among the age categories. This is consistent with other studies showing better outcomes among 5–9 years olds compared to other child and adolescent age groups [23].

Even though we found a high rate of favourable outcomes amongst children and adolescents treated for DR-TB, almost 10% of the patients died while on treatment. This along with the fact that most children and adolescents develop primary DR-TB points towards a need for preventive treatment. We have shown previously that a fluoroquinolone-based preventive treatment for presumed DR-TB infection amongst household contacts of DR-TB patients in our setting is safe and 65% effective [30]. Scaling up the use of DR-TB preventive treatment can decrease the burden of DR-TB disease amongst children and adolescents. 

Limitations of this study include not all children being confirmed with DR-TB. We had 14 (5.3%) children in our analysis, who were household contacts of individuals diagnosed with DR-TB and among whom microbiological confirmation could not be obtained. Studies have shown a high level of concordance for isoniazid/rifampicin resistance profile among close contacts of DR-TB index patients so we assumed that the child contacts had a similar resistance profile as the index patient’s strain and treated accordingly [31]. As we used programmatic surveillance data, information on nutritional surveillance, the severity of disease and adherence to drugs was not available. This study also does not analyze outcomes by different drug regimens in use at the program. The strengths of our study include the inclusion of a significant proportion of children nationally reported from Pakistan, an outcome analysis of the 15–19 year adolescent category, successful programmatic management of DR-TB in most and the availability of complete medical records for analysis. 

Overall, we found high rates of favourable outcomes in children and adolescents treated for DR-TB at a tertiary care center in Pakistan. We also found a significant difference by gender with males being underrepresented and experiencing worse outcomes compared to females. A large proportion of those treated for DR-TB in our cohort was 15 years or older and we are using this opportunity to highlight this important group. Globally, TB in adolescents along with outcomes needs to be accounted for and studied in order to detect gaps in care and plan for and implement age-specific services. More specific to our program, we need urgent research to elucidate reasons for poor outcomes amongst males and to develop context-specific strategies to address our programmatic gaps. 

## Figures and Tables

**Table 1:** Baseline drug resistance and its impact on treatment outcome observed amongst child and adolescent TB patients at a tertiary care hospital in Karachi, Pakistan between January 2014 and December 2019.

Resistance Type	*n* (col%) (N = 262)	Favourable Outcome *n* (row%)
RR/MDR-TB	198 (75.6)	150 (75.8)
PreXDR-TB	43 (16.4)	30 (69.8)
XDR-TB	3 (1.2)	2 (66.7)
Other	4 (1.5)	3 (75.0)
Suspected DR-TB	14 (5.3)	9 (64.3)

**Table 2:** DR-TB Treatment outcomes by age groups observed amongst child and adolescent TB patients at a tertiary care hospital in Karachi, Pakistan between January 2014 and December 2019 (N = 262).

Age	*n* (col%) (N = 262)	Favourable Outcomes *n* (row%)
0–4 y	16 (6.1)	11 (68.8)
5–9 y	10 (3.8)	8 (80.0)
10–14 y	54 (20.6)	40 (74.1)
15–19 y	182 (69.5)	135 (74.2)

Chi-square *p*-value = 0.94.

## Data Availability

Data access can be requested from corresponding author.

## References

[1] World Health Organization (2020). Global Tuberculosis Report 2020.

[2] Dodd P.J., Sismanidis C., Seddon J.A. (2016). Global burden of drug-resistant tuberculosis in children: A mathematical modelling study. Lancet Infect. Dis..

[3] Jenkins H.E., Tolman A.W., Yuen C.M., Parr J.B., Keshavjee S., Pérez-Vélez C.M., Pagano M., Becerra M.C., Cohen T. (2014). Incidence of multidrug-resistant tuberculosis disease in children: Systematic review and global estimates. Lancet.

[4] Song W.M., Li Y.F., Liu Y.X., Liu Y., Yu C.B., Liu J.Y., Li H.C. (2021). Drug-Resistant Tuberculosis Among Children: A Systematic Review and Meta-Analysis. Front. Public Health.

[5] Malik A.A., Gandhi N.R., Marcy O., Walters E., Tejiokem M., Chau G.D., Omer S.B., Lash T.L., Becerra M.C., Njuguna I.N. (2022). Development of a clinical prediction score including monocyte-to-lymphocyte ratio to inform tuberculosis treatment among children with HIV: A multi-country study. Open Forum. Infect. Dis..

[6] Wood R., Liang H., Wu H., Middelkoop K., Oni T., Rangaka M.X., Wilkinson R.J., Bekker L.G., Lawn S.D. (2010). Changing prevalence of tuberculosis infection with increasing age in high-burden townships in South Africa. Int. J. Tuberc. Lung Dis..

[7] Seddon J.A., Chiang S.S., Esmail H., Coussens A.K. (2018). The Wonder Years: What Can Primary School Children Teach Us About Immunity to Mycobacterium tuberculosis?. Front. Immunol..

[8] Snow K.J., Cruz A.T., Seddon J.A., Ferrand R.A., Chiang S.S., Hughes J.A., Kampmann B., Graham S.M., Dodd P.J., Houben R.M. (2020). Adolescent tuberculosis. Lancet Child Adolesc. Health.

[9] Nahid P., Mase S.R., Migliori G.B., Sotgiu G., Bothamley G.H., Brozek J.L., Cattamanchi A., Cegielski J.P., Chen L., Daley C.L. (2019). Treatment of Drug-Resistant Tuberculosis. An Official ATS/CDC/ERS/IDSA Clinical Practice Guideline. Am. J. Respir. Crit. Care Med..

[10] National TB Control Program (2019). Annual Report 2019.

[11] Migliori G.B., Network G.T. (2018). Evolution of Programmatic Definitions Used in Tuberculosis Prevention and Care. Clin. Infect. Dis..

[12] Harausz E.P., Garcia-Prats A.J., Law S., Schaaf H.S., Kredo T., Seddon J.A., Menzies D., Turkova A., Achar J., Amanullah F. (2018). Treatment and outcomes in children with multidrug-resistant tuberculosis: A systematic review and individual patient data meta-analysis. PLoS Med..

[13] Tola H.H., Khadoura K.J., Jimma W., Nedjat S., Majdzadeh R. (2020). Multidrug resistant tuberculosis treatment outcome in children in developing and developed countries: A systematic review and meta-analysis. Int. J. Infect. Dis..

[14] Dhakulkar S., Das M., Sutar N., Oswal V., Shah D., Ravi S., Vengurlekar D., Chavan V., Rebello L., Meneguim A.C. (2021). Treatment outcomes of children and adolescents receiving drug-resistant TB treatment in a routine TB programme, Mumbai, India. PLoS ONE.

[15] Abubakar M., Ahmad N., Atif M., Hayat Khan A., Ghafoor A. (2022). Treatment outcomes among childhood extensively drug-resistant tuberculosis patients in Pakistan. ERJ Open Res..

[16] Naz F., Ahmad N., Wahid A., Ahmad I., Khan A., Abubakar M., Khan S.A., Khan A., Latif A., Ghafoor A. (2021). High rate of successful treatment outcomes among childhood rifampicin/multidrug-resistant tuberculosis in Pakistan: A multicentre retrospective observational analysis. BMC Infect. Dis..

[17] Isaakidis P., Paryani R., Khan S., Mansoor H., Manglani M., Valiyakath A., Saranchuk P., Furin J. (2013). Poor outcomes in a cohort of HIV-infected adolescents undergoing treatment for multidrug-resistant tuberculosis in Mumbai, India. PLoS ONE.

[18] Moyo S., Furin J.J., Hughes J., Daniels J., Snyman L., Muller O., Cox V., Shroufi A., Cox H. (2015). Outcomes in Adolescents Undergoing Treatment for Drug-Resistant Tuberculosis in Cape Town, South Africa, 2008–2013. Arch. Pediatr. Infect. Dis..

[19] Hamid M., Brooks M.B., Madhani F., Ali H., Naseer M.J., The Childhood Tuberculosis Karachi G., Becerra M., Amanullah F. (2019). Risk factors for unsuccessful tuberculosis treatment outcomes in children. PLoS ONE.

[20] Codlin A.J., Khowaja S., Chen Z., Rahbar M.H., Qadeer E., Ara I., McCormick J.B., Fisher-Hoch S.P., Khan A.J. (2011). Short report: Gender differences in tuberculosis notification in Pakistan. Am. J. Trop. Med. Hyg..

[21] Chiang S.S., Dolynska M., Rybak N.R., Cruz A.T., Aibana O., Sheremeta Y., Petrenko V., Mamotenko A., Terleieva I., Horsburgh C.R. (2020). Clinical manifestations and epidemiology of adolescent tuberculosis in Ukraine. ERJ Open Res..

[22] Laycock K.M., Enane L.A., Steenhoff A.P. (2021). Tuberculosis in Adolescents and Young Adults: Emerging Data on TB Transmission and Prevention among Vulnerable Young People. Trop. Med. Infect. Dis..

[23] Osman M., du Preez K., Seddon J.A., Claassens M.M., Dunbar R., Dlamini S.S., Welte A., Naidoo P., Hesseling A.C. (2021). Mortality in South African Children and Adolescents Routinely Treated for Tuberculosis. Pediatrics.

[24] Tierney D.B., Milstein M.B., Manjourides J., Furin J.J., Mitnick C.D. (2016). Treatment Outcomes for Adolescents With Multidrug-Resistant Tuberculosis in Lima, Peru. Glob. Pediatr. Health.

[25] Chiang C.Y., Bai K.J., Lee C.N., Enarson D.A., Suo J., Luh K.T. (2010). Inconsistent dosing of anti-tuberculosis drugs in Taipei, Taiwan. Int. J. Tuberc. Lung Dis..

[26] Enane L.A., Lowenthal E.D., Arscott-Mills T., Matlhare M., Smallcomb L.S., Kgwaadira B., Coffin S.E., Steenhoff A.P. (2016). Loss to follow-up among adolescents with tuberculosis in Gaborone, Botswana. Int. J. Tuberc. Lung Dis..

[27] Stevens H., Ximenes R.A., Dantas O.M., Rodrigues L.C. (2014). Risk factors for tuberculosis in older children and adolescents: A matched case-control study in Recife, Brazil. Emerg. Themes Epidemiol..

[28] Moore B.K., Anyalechi E., van der Walt M., Smith S., Erasmus L., Lancaster J., Morris S., Ndjeka N., Ershova J., Ismail N. (2015). Epidemiology of drug-resistant tuberculosis among children and adolescents in South Africa, 2005 –2010. Int. J. Tuberc. Lung Dis..

[29] Das M., Mathur T., Ravi S., Meneguim A.C., Iyer A., Mansoor H., Kalon S., Hossain F.N., Acharya S., Ferlazzo G. (2021). Challenging drug-resistant TB treatment journey for children, adolescents and their care-givers: A qualitative study. PLoS ONE.

[30] Malik A.A., Gandhi N.R., Lash T.L., Cranmer L.M., Omer S.B., Ahmed J.F., Siddiqui S., Amanullah F., Khan A.J., Keshavjee S. (2021). Effectiveness of Preventive Therapy for Persons Exposed at Home to Drug-Resistant Tuberculosis, Karachi, Pakistan. Emerg. Infect. Dis..

[31] Chiang S.S., Brooks M.B., Jenkins H.E., Rubenstein D., Seddon J.A., van de Water B.J., Lindeborg M.M., Becerra M.C., Yuen C.M. (2021). Concordance of Drug-resistance Profiles Between Persons With Drug-resistant Tuberculosis and Their Household Contacts: A Systematic Review and Meta-analysis. Clin. Infect. Dis..

